# 

**Published:** 1827-09

**Authors:** 


### MONTHLY LIST OF MEDICAL BOOKS.

*[No books can be entered on this List except those sent to us for the purpose; as, in the list hitherto transmitted, the names of works have frequently been given as published, which have not appeared for weeks, or even months, after.]*

Practical Observations on the Management and Diseases of Children. By the late T. C. Haden, Esq. With additional Observations, and a Biographical Notice of the Author, by Thomas Alcock, Surgeon.

The Medical and Surgical Student's Synopsis and Guide; comprising a Detail of the proper Course of Study, and Works to be consulted, &c. By G. T. HAYDEN, Licentiate of the Royal College of Surgeons in Ireland.—12mo. pp. 141. Hodges and M'Arthur, Dublin.

Mems. Maxims, and Memoirs. By WILLIAM WADD, Esq. F.L.S. Surgeon Extraordinary to the King, &c. &c. &c.—8vo. pp. 303; with Plates. Callow and Wilson, London, 1827.

Medical Ethics; or, a Code of Institutes and Precepts adapted to the Professional Conduct of Physicians and Surgeons. By the late THOMAS PERCIVAL, M.D. F.R.S and A.S. Lond. F.R.S. and R.M.S. Edinb. &c. &c. With Additions, illustrative of the past and present State of the Profession, and its Collegiate Institutions, in Great Britain.—12mo. pp. 360. W. Jackson, London, 1827.

### METEOROLOGICAL JOURNAL,

### NOTICES.

Mr. C. *will perceive that he mistook the cause of the delay to which he alludes. We shall be most happy to hear from him again.*

*Papers are preparing for the Press from* Mr. BRODIE, Mr. BABINGTON, Mr. ROSE, Dr. M. HALL, Mr. SERPH, *and* Mr. FROST.

*The letters of* Mr. BUSH *and* Mr. TAYLOR *came to hand just as we were going to press.*

